# Bedside measurement of microcirculation in critically ill patients: A scoping review protocol

**DOI:** 10.1371/journal.pone.0341435

**Published:** 2026-02-05

**Authors:** Sogand Sarmadi, Neda Sanaie, Fateme Monjazebi, Akbar Zare-Kaseb

**Affiliations:** 1 Department of Medical-Surgical Nursing, School of Nursing and Midwifery, Shahid Beheshti University of Medical Sciences, Tehran, Iran; 2 Student Research Committee, Department of Medical-Surgical Nursing, School of Nursing and Midwifery, Shahid Beheshti University of Medical Sciences, Tehran, Iran; Klinikum Fulda gAG, GERMANY

## Abstract

**Background:**

Microcirculatory dysfunction is a pivotal factor in the pathophysiology and prognosis of critically ill patients. While traditional resuscitation primarily emphasizes macrocirculatory parameters such as mean arterial pressure (MAP), emerging evidence indicates that maintaining a normal MAP alone does not guarantee adequate microvascular perfusion. As a result, bedside evaluation of microcirculation has garnered increased interest as a supplementary method to hemodynamic monitoring.

**Objective:**

This scoping review aims to systematically map and categorize existing bedside tools and technologies used to assess microcirculation in critically ill patients. The review will summarize their clinical applications, advantages, limitations, and integration into intensive care practice, and identify key evidence gaps to guide future research.

**Methods:**

In accordance with the Joanna Briggs Institute (JBI) framework and the PRISMA-ScR guidelines, a comprehensive literature search will be conducted across MEDLINE, Scopus, Web of Science, Embase, CINAHL, the Cochrane Library, and ProQuest. Additionally, the grey literature search will be complemented by searches of trial registries such as ClinicalTrials.gov and the WHO ICTRP. Eligible studies will encompass all primary research involving critically ill patients evaluated for microcirculatory function at the bedside within intensive care units (ICUs). Data extraction and synthesis will adhere to an inductive–deductive thematic approach based on Braun and Clarke’s methodology, complemented by the PAGER framework to identify patterns, advances, gaps, and evidence pertinent to clinical practice.

**Conclusion:**

By mapping the landscape of bedside microcirculatory assessment tools, this review will support the translation of microcirculatory monitoring from research to clinical practice, thereby promoting physiologically guided, individualized management in critically ill patients.

**Registration:**

This protocol is prospectively registered in OSF.io.

## Introduction

The resuscitation of circulation in critically ill patients involves the restoration of sufficient oxygen supply, tissue perfusion, and, ultimately, cellular metabolism [1]. The main function of the circulatory system is to maintain sufficient blood flow, enabling oxygen and nutrients to reach tissues and waste products to be removed [2]. Achieving this requires generating sufficient perfusion pressure to promote blood flow into the capillaries [3].

Historically, clinical decisions relied on the assessment of blood pressure, cardiac output, and other macrocirculatory indicators. Mean arterial pressure (MAP) has emerged as a widely utilized metric for assessing tissue and organ perfusion [4]. Recent evidence shows no correlation between MAP and blood flow adequacy in the microcirculation of end capillaries. Furthermore, research has demonstrated that microcirculatory dysfunction may persist despite maintaining normal MAP [5,6].

Blood lactate concentration is a biomarker for assessing tissue perfusion and oxygenation. It seems to result from anaerobic glycolysis caused by insufficient oxygenation and tissue hypoxia [7,8]. However, the common understanding of lactate as an essential byproduct of anaerobic metabolism and a sign of cellular distress cannot capture the complexity of the processes involved. Our cells constantly generate and consume lactate in significant amounts, even under fully aerobic conditions. While marked hyperlactatemia is always a red flag in our patients, it does not always show a life-threatening condition. Similarly, not all critically ill patients show hyperlactatemia [9,10].

The ultimate component of the cardiovascular system is microcirculation. Microcirculation is a diverse and intricate network comprising arterioles, capillaries, and venules that connect the arterial and venous systems. Its primary role is to meet the metabolic needs of tissues, accomplished through regulating organ perfusion and oxygen distribution [11]. Several studies have emphasized that evaluating microcirculation is crucial alongside macrocirculation, as it can address deficiencies in microvascular function [12,13].

Given its physiological importance, the routine evaluation of microcirculation in intensive care units (ICUs) has been strongly advocated. However, despite technological advances, bedside assessment of microcirculation remains absent, mainly in routine clinical practice [14]. Therefore, a comprehensive synthesis of the available evidence is needed to identify, categorize, and evaluate existing bedside tools and techniques for assessing microcirculation in critically ill patients. The present scoping review aims to map the range of currently available methods, summarize their potential benefits and limitations, and outline key gaps and priorities to guide future research and clinical implementation.

## Methods

### Design

**Fig 1:**
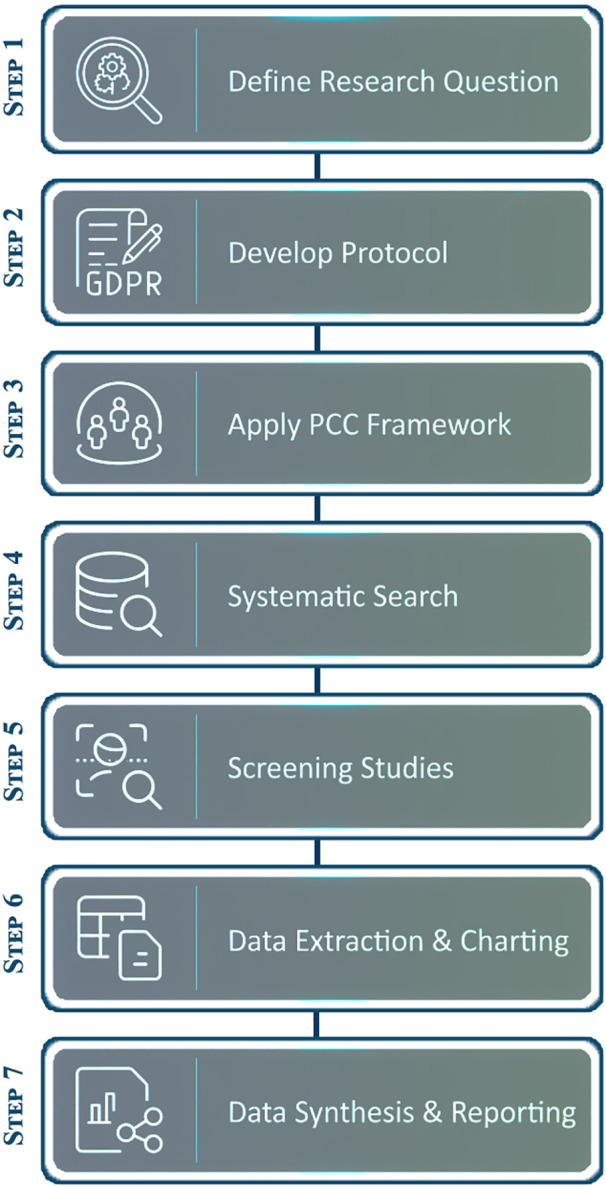
Systematic research workflow for microcirculatory assessment tools.

Our methodological approach is well-suited to the goals of this study. Scoping reviews—especially those conducted using Joanna Briggs Institute (JBI) methods—are meant to map concepts, identify patterns and developments, and highlight gaps in the literature, thereby clarifying what evidence is needed to improve practice [15–17]. They are particularly beneficial when a phenomenon has not been extensively studied and when the aim is to synthesize and disseminate the existing research to inform recommendations for future investigations [15,17]. To enhance methodological rigor and ensure transparency in reporting, this scoping review will adhere to the established seven methodical stages provided by the JBI ((1) defining the research question, (2) developing the protocol, (3) applying the Population/Concept/Context (PCC) framework, (4) performing systematic searches, (5) screening studies, (6) extracting and charting relevant data, and (7) synthesizing and reporting the evidence) [15–17] (Fig 1) and the PRISMA family of reporting standards. We reported the protocol in accordance with PRISMA-P (S1 Appendix) [18], and the final review will be reported in accordance with the PRISMA Extension for Scoping Reviews (PRISMA-ScR) [18–21]. Furthermore, we will employ the PAGER framework (Patterns, Advances, Gaps, Evidence for Practice, and Research recommendations) to enhance the processes of charting, analysis, synthesis, and presentation of findings, as PAGER offers practical guidance on data charting and synthesis that complements the PRISMA-ScR methodology [22]. Additionally, the methodological framework will combine Braun and Clarke’s inductive-deductive approach [23].

### Main review question

What bedside tools and technologies have been employed to evaluate microcirculation in critically ill patients within ICUs?

### Sub-questions

1. What types of bedside methods and indicators are available for evaluating microcirculatory function in critically ill patients?
2. What are the advantages, limitations, and clinical applications of each tool in ICUs?
3. How have these tools been validated or integrated into routine critical care monitoring?
4. How have clinicians been involved in applying or evaluating these tools in research or clinical practice?

### Search strategy

The search strategy will be comprehensive and systematic. The following electronic databases will be searched: MEDLINE (via PubMed), Web of Science, Scopus, Embase, CINAHL, Cochrane Library, and ProQuest, as well as Google Scholar. All records from the inception of each database through the date of the search will be included. If the review process extends over time, an updated search will be conducted to identify newly published studies.

To ensure the search is as comprehensive as possible and minimize the risk of missing relevant evidence, the reference lists of all included studies and related review articles will be screened, and expert opinions will be consulted as needed. Furthermore, trial registries will be searched, including ClinicalTrials.gov, the WHO International Clinical Trials Registry Platform (ICTRP), the Japan Primary Registries Network (JPRN), and the Iranian Registry of Clinical Trials (IRCT). These registries will be examined to identify ongoing, unpublished, or recently completed studies relevant to the topic.

The search will integrate controlled vocabulary terms (MeSH and Emtree) with free-text keywords. The summary of the search terms includes: “Microcirculation”, “Microvascular Blood Flow”, “Microvascular Circulation”, “Intensive Care Unit”, “Hemodynamics”, “Point-of-care”, “Clinical Parameter”, and “Evaluation Tool.”

Boolean operators AND and OR will be used to appropriately connect terms: synonyms within each concept will be combined with OR, and distinct conceptual components will be linked using AND. The search strategy will be refined iteratively using MeSH and Emtree indexing, expert consultation, and free-text approaches to optimize sensitivity and specificity for identifying studies relevant to bedside measurement of microcirculation in critically ill patients.

### Eligibility criteria

The Population–Concept–Context (PCC) framework recommended by JBI serves as the guiding methodology for this scoping review (Table 1).

**Table 1:** The population–concept–context (PCC) framework to formulate the review question.

Component	Description	Examples/ Notes
**Population (P)**	Critically ill patients receiving intensive or critical care, regardless of underlying condition (sepsis, septic shock, trauma, cardiac surgery, multi-organ failure).	Studies including ICU or critical care populations.
**Concept (C)**	Bedside tools, devices, or clinical parameters used to assess microcirculation or tissue perfusion in critically ill patients.	Includes both direct and indirect methods such as: capillary refill time, mottling score, skin temperature, near-infrared spectroscopy (NIRS), contrast-enhanced ultrasound (CEUS), handheld vital microscopy (HVM), laser Doppler flowmetry, nailfold capillaroscopy, Pv–aCO₂ gap, and plethysmography.
**Context (C)**	Intensive care or critical care settings (ICUs or emergency units that provide organ support).	Studies conducted in hospital-based acute care settings; excludes outpatient or prehospital studies.

The population (P) of interest encompasses critically ill adult, pediatric, and neonatal patients who are admitted to critical care units due to conditions such as sepsis, septic shock, trauma, cardiac surgery, or multi-organ failure.

The concept (C) focuses on bedside tools, devices, and clinical parameters used to assess microcirculation or tissue perfusion in critically ill patients. This encompasses both direct and indirect assessment methods, including but not limited to capillary refill time (CRT), mottling score, skin temperature gradients, near-infrared spectroscopy (NIRS), contrast-enhanced ultrasound (CEUS), handheld vital microscopy (HVM), laser Doppler flowmetry, nailfold capillaroscopy, veno-arterial CO₂ gap (Pv–aCO₂ gap), and plethysmography.

The context (C) of this review includes intensive or critical care settings, such as ICUs, high-dependency units (HDUs), or emergency departments (EDs), that provide organ support.

This review will encompass all primary research studies, including both quantitative and qualitative investigations, irrespective of their publication status. The full text must be accessible to the authors. If institutional access is unavailable, the corresponding author will be contacted via email up to three times. Authors will be granted a one-month response period, with the deadline explicitly noted in all communications. A follow-up email will be dispatched two weeks after the initial contact, and a third attempt will be made one month subsequently. The inclusion and exclusion criteria are delineated in Table 1.

All types of review articles will be excluded from this study. In addition, studies conducted exclusively on animal populations or those conducted in non-acute or outpatient care settings will be excluded.

### Screening and selection

The web-based HubMeta platform will facilitate the main review tasks, such as automatic removal of duplicates, screening titles and abstracts, full-text review, and data extraction. In the initial phase, two reviewers, A.Z. and S.S., will evaluate titles and abstracts to determine eligibility. A pilot test using a random sample of 25 articles will be conducted, employing predefined criteria to enhance screening consistency. Subsequently, the kappa statistic will be utilized to assess the level of agreement between reviewers concerning study inclusion [24]. Kappa statistics will be interpreted using standard thresholds: 0 or below indicating no agreement, 0.01–0.20 as slight, 0.21–0.40 as fair, 0.41–0.60 as moderate, 0.61–0.80 as substantial, and 0.81–1.00 as almost perfect agreement. For this review, only studies with kappa values greater than 0.8, indicating strong agreement, will be included [25]. Any disagreements will be resolved through discussion and, if necessary, by involving more experienced reviewers (N.S. and F.M.).

Full-text articles of all relevant and potentially relevant studies will be independently retrieved and assessed by two reviewers (A.Z. and S.S.), with any disagreements resolved by a third reviewer (N.S.). Studies that do not meet the inclusion criteria will be excluded. To enhance feasibility, ensure proper use of the data collection tools, and identify possible gaps or issues in the scoping review protocol, a pilot test will be conducted on the first 25 studies. This step will help the research team become familiar with the protocol procedures and enable refinement of the inclusion and exclusion criteria to ensure consistent application. Detailed reasons for excluding studies will be recorded after full-text evaluation, and a summary of excluded studies, along with their references and the rationale for exclusion, will be provided. A PRISMA flowchart illustrates the entire screening process.

### Data extraction

During data extraction, a standardized charting form will be used and piloted on a limited sample of articles before full implementation. Two reviewers will independently complete the form, and any discrepancies will be addressed through discussion or consultation with a third reviewer.

The extracted items shall encompass study identification details (including author(s), year of publication, country, and source), study design and methodological characteristics (such as the type of study, sample size, geographic or clinical setting, and ICU type), population characteristics and the clinical context (including age group, inclusion criteria, type of critical illness such as sepsis or shock, and ICU category), as well as comprehensive information regarding the bedside tool or method employed for microcirculatory assessment (including the name of the tool, its classification—whether clinical, optical, ultrasound-based, imaging, or biochemical—measurement principles, device features such as invasiveness, point-of-care or continuous mode, operator requirements, and the software utilized).

Extracted parameters will also include the variables or indices measured (StO₂, MFI, PVD, PI, capillary refill time, Pv–aCO₂ gap, lactate), the purpose of use (monitoring, diagnosis, prognostic prediction, or assessment of therapeutic response), and main results and key findings (including correlations with clinical indicators or patient outcomes). Furthermore, the authors’ advantages and limitations will be documented (bedside feasibility, accuracy, cost, training requirements, and sensitivity to pigmentation or temperature).

## Risk of Bias

While critical appraisal is not required in scoping reviews, an optional quality assessment will be carried out to evaluate the strength and reliability of the evidence. This assessment aims not to exclude studies but to summarize their overall quality and identify possible biases in the included literature.

Two reviewers will independently evaluate the methodological quality of each included study using the most appropriate JBI critical appraisal tools, selected according to study design (cross-sectional, cohort, randomized controlled trial, or qualitative study) [26]. The assessment will consider key domains, including clarity of objectives, sampling strategy, data collection procedures, validity and reliability of measurement tools, completeness of outcome reporting, and potential sources of bias (selection, measurement, or reporting). Any disagreements between reviewers will be resolved through discussion by a third reviewer.

The results of the risk-of-bias assessment will be presented descriptively and summarized in tabular form to provide a transparent overview of the methodological robustness of the included studies. The quality appraisal will support the interpretation of the findings, but will not serve as a criterion for study exclusion.

### Data synthesis

Findings will primarily be summarized using descriptive statistics, including frequencies and percentages, medians with interquartile ranges, and overall minimum-to-maximum ranges. Tables and figures will be employed to illustrate the characteristics of the included studies, while the narrative synthesis in the text will provide additional contextual information.

For data synthesis, a descriptive and thematic analysis will be conducted in accordance with the JBI guidelines for scoping reviews. Extracted data will first be organized into summary tables to provide an overview of study characteristics, populations, and the bedside tools or methods used for microcirculatory assessment.

Subsequently, the findings will be synthesized employing an inductive–deductive thematic approach, adapted from Braun and Clarke’s framework, to identify recurring patterns, concepts, and gaps within the evidence base. This analysis will delineate how various bedside tools have been developed, validated, and implemented in ICUs. It will underscore similarities and differences among measurement methodologies (clinical parameters, imaging-based techniques, optical spectroscopy, and biochemical indicators).

Themes will also be generated to capture methodological trends, reported advantages and limitations, and implications for clinical practice and future research. Where possible, tools will be categorized by their purpose (diagnostic, monitoring, or prognostic), measurement principles, and feasibility at the bedside.

The synthesis will produce a conceptual framework that summarizes existing evidence on bedside microcirculatory assessment in critically ill patients, identifies significant research gaps, and suggests potential directions for future innovation and implementation in critical care monitoring.

### Timeline of the study

The anticipated timeline for the different phases of the study is as follows:

- Record Identification and Importing: December 2025 – January 2026
- Title and Abstract Screening: January – February 2026
- Full-Text Screening: February – March 2026
- Data Extraction: March – April 2026
- Data Analysis and Synthesis: April – May 2026
- Manuscript Drafting of the Scoping Review Findings: May 2026

Therefore, the results of the full scoping review are expected to be ready by May 2026.

### Ethics consideration

This study is a scoping review protocol and involves the analysis of previously published studies only. No primary data collection from human participants or animals will be conducted. Therefore, formal ethical approval and informed consent were not required. The findings of this study will be disseminated through academic publications and conference presentations.

## Discussion

Previous evaluations have consistently demonstrated that assessing the microcirculation, in conjunction with macrocirculatory evaluation, is essential for the management of critically ill patients. This is attributed to the fact that macro-hemodynamic parameters, such as MAP, do not necessarily correspond to the adequacy of microvascular blood flow, and microcirculatory dysfunction may endure even when MAP is within normal limits [27].

Previous assessments have documented various methodologies for bedside evaluation of microcirculation, including HVM, NIRS, laser Doppler flowmetry, and clinical indicators such as CRT and mottling score. Each of these techniques possesses distinct technical benefits and limitations. The abundance of available methods has led to fragmented and heterogeneous evidence; consequently, our scoping review intends to systematically delineate the tools, parameters, and clinical applications of these technologies [28,29].

Evidence suggests that emerging methods, such as sublingual microcirculatory assessment using handheld microscopes coupled with automated image analysis software, show significant promise as point-of-care modalities. These technologies can provide real-time visualization of capillary perfusion and may offer a more immediate, cost-effective approach to bedside patient monitoring [30,31].

Despite their potential, most microcirculatory assessment techniques remain research tools rather than standard clinical instruments. The predominant barriers include methodological heterogeneity across studies, the absence of universally accepted cutoff values or clinical thresholds, technical limitations such as motion artifacts and reliance on skin pigmentation or temperature, operator training requirements, and the lack of large-scale RCTs demonstrating improved patient outcomes. In essence, there remains limited high-quality evidence indicating that direct targeting of microcirculatory parameters leads to improved survival or organ function [32,33].

Numerous reviews have emphasized the critical necessity to establish standardized imaging acquisition protocols, reporting metrics, and analytical methodologies. In the absence of harmonized procedures and validated analytical instruments, it becomes challenging to compare outcomes across different studies or to synthesize evidence effectively. This highlights the significance of conducting a scoping review to identify methodological gaps and to inform the development of standardized approaches for subsequent research [30,34].

Throughout the literature, significant research gaps have been identified, including the paucity of sufficiently powered RCTs comparing microcirculation-guided therapy with standard care; the absence of standardized outcome measures and reporting guidelines; limited evidence concerning specific subpopulations such as the elderly or patients with non-septic shock; and the lack of data on cost-effectiveness and implementation feasibility. Mapping these gaps through this scoping review will help prioritize future research directions [35,36].

For the successful clinical translation of microcirculatory monitoring, various concurrent efforts are required: the development of standardized operating procedures and validated indices; structured operator training programs to ensure interobserver reliability; implementation studies to evaluate clinical workload and acceptability; and research assessing how microcirculatory data can inform therapeutic decisions and enhance measurable clinical outcomes [31,37].

### Limitations

This scoping review will be constrained by the heterogeneity of study designs, patient populations, and outcome measures across the included literature. As the review aims to map rather than critically evaluate evidence, no meta-analysis will be conducted. Furthermore, limiting the search to clinical and technological assessment instruments may introduce variability in measurement methods and reporting standards.

## Conclusion

This scoping review will systematically map the current evidence regarding bedside tools and technologies used to assess microcirculation in critically ill patients. By summarizing the range, applications, and limitations of these methods, the review will highlight critical gaps in evidence and opportunities for future innovation in microcirculatory monitoring. The findings are expected to inform clinicians, researchers, and policymakers on the feasibility and integration of microcirculatory assessment into critical care practice, ultimately contributing to more individualized, physiologically guided patient management in ICUs.

## Supporting information

S1 Appendix The PRISMA-P checklist. (DOCX)
